# Retraction: Prevalence, correlates of undernutrition and intestinal parasitic infection among children below 5 years living in the forest community of Ndelele, East Region of Cameroon: A cross-sectional assessment

**DOI:** 10.1371/journal.pone.0333989

**Published:** 2025-10-07

**Authors:** The PLOS One Editors

After this article [1] was published, the authors contacted the *PLOS One* Editors to report errors in the data analysis. Specifically,

- The analyses reported in [1] were carried out with a sample size of 422; however, it was later identified that 182 samples should have been removed, as follows:
  - 118 Household IDs that did not match/were not paired between the nutrition and infection sections
  - 58 observations for which the child’s age was not within the range of 12-59 months
  - 6 samples for which the child was absent during the nutrition survey

Consequently, the correct overall sample size is 240.

- There was an error in calculation of nutritional status because the standard WHO categorization was not used. When the standard WHO categorization is used, the authors reported that 49.1% of children were classified as undernourished and 3.8% were classified as wasting compared to the originally reported values in [1] of 83.77% and 28.64%, respectively.

The authors provided the results of the reanalyses using the corrected data, noting that several of the results reported in [1] are no longer supported.

The changes reported by the authors were assessed by a statistical reviewer and a member of the *PLOS One* Editorial Board, who advised that as a consequence of the reduced sample size, the study is underpowered and the conclusions are not supported.

In light of the above concerns, the *PLOS One* Editors retract this article.

SMG responded but expressed neither agreement nor disagreement with the editorial decision. BFA, CMS, RAS, MTA, NS, AY, EK, AI, CYT, and EA either did not respond directly or could not be reached.
